# Supplementary material for: Role of Menopausal Transition and Physical Activity in Loss of Lean and Muscle Mass: A Follow-Up Study in Middle-Aged Finnish Women
Source: J Clin Med. 2020 May 23;9(5):1588. doi: 10.3390/jcm9051588 (PMC7290663; doi:10.3390/jcm9051588)
Supplement: Supplementary file 1 [file jcm-09-01588-s001.zip › S1_JCM.docx]

**Table S1.** Characteristics of the participants who used progestogen-based contraception at baseline.

|  | Baseline  n = 73 | Final follow-up  n = 73 | Difference  % | *P* |
| --- | --- | --- | --- | --- |
| Age, y | 51.0 ± 1.7 | 52.4 ± 1.8 | **+2.6** | **<0.001^a^** |
| Body mass, kg | 68.2 ± 10.6 | 68.5 ± 10.8 |  | 0.245^b^ |
| BMI, kg/m^2^ | 24.9 ± 3.7 | 25.0 ± 3.9 |  | 0.234^b^ |
| E_2_, nmol/L | 0.38 ± 0.34 | 0.24 ± 0.18 | **-37** | **0.002^b^** |
| FSH, IU/L | 35.1 ± 22.2 | 64.0 ± 27.5 | **+82** | **<0.001**^b^ |
| Physical activity |  |  |  |  |
| MVPA, min/day^X^ (n = 47) | 51.8 ± 25.5 | 45.9 ± 20.3 |  | 0.120^b^ |
| MET-hours/day^XX^ (n = 70) | 5.0 ± 4.2 | 4.8 ± 3.7 |  | 0.791^b^ |
| DXA-measurements |  |  |  |  |
| LBM, kg (n = 69) | 42.4 ± 4.5 | 42.3 ± 4.4 |  | 0.548^a^ |
| LBMI, kg/m^2^ (n = 69) | 15.4 ± 1.3 | 15.4 ± 1.3 |  | 0.561^a^ |
| ALM, kg (n = 69) | 18.3 ± 2.0 | 18.2 ± 2.0 |  | 0.416^a^ |
| ALMI, kg/m^2^ (n = 69) | 6.7 ± 0.6 | 6.6 ± 0.5 |  | 0.397^a^ |
| Right leg lean mass, kg (n = 69) | 6.9 ± 0.7 | 6.9 ± 0.8 |  | 0.712^a^ |

Values are given as mean ± SD. ALM, appendicular lean mass; ALMI, appendicular lean mass index; BMI, body mass index; E_2,_ estradiol; FSH, follicle stimulating hormone; LBM, lean body mass; LBMI, lean body mass index; MET, metabolic equivalent; MVPA, moderate-to-vigorous physical activity. ^a^ paired t-test, ^b^ Wilcoxon Signed rank test, ^X^ accelerometer-measured, ^XX^ self-reported. Significant results (*P* ≤ 0.050) are shown in bold.
