# Supplementary material for: Role of Menopausal Transition and Physical Activity in Loss of Lean and Muscle Mass: A Follow-Up Study in Middle-Aged Finnish Women
Source: J Clin Med. 2020 May 23;9(5):1588. doi: 10.3390/jcm9051588 (PMC7290663; doi:10.3390/jcm9051588)
Supplement: Supplementary file 1 [file jcm-09-01588-s001.zip › S2_JCM.docx]

**Table S2**. Characteristics of the participants who did not use progestogen-based contraception at baseline.

|  | Baseline  n = 161 | Final follow-up  n = 161 | Difference  % | *P* |
| --- | --- | --- | --- | --- |
| Age, y | 51.8 ± 2.0 | 53.2 ± 1.9 | **+2.7** | **<0.001^b^** |
| Body mass, kg | 70.5 ± 11.5 | 71.3 ± 11.8 | **+1.1** | **<0.001^b^** |
| BMI, kg/m^2^ | 25.9 ± 4.0 | 26.2 ± 4.2 | **+1.1** | **<0.001^b^** |
| E_2_, nmol/L | 0.32 ± 0.23 | 0.24 ± 0.19 | **-25** | **<0.001^b^** |
| FSH, IU/L | 36.5 ± 21.3 | 68.2 ± 28.3 | **+87** | **<0.001**^b^ |
| Physical activity |  |  |  |  |
| MVPA, min/day^X^ (n = 126) | 51.8 ± 30.7 | 51.1 ± 24.7 |  | 0.753^b^ |
| MET-hours/day^XX^ (n = 161) | 4.2 ± 3.8 | 4.6 ± 3.6 | **+9.7** | **0.027^b^** |
| DXA-measurements |  |  |  |  |
| LBM, kg (n = 154) | 41.4 ± 4.3 | 41.1 ± 4.3 | **-0.6** | **0.018^a^** |
| LBMI, kg/m^2^ (n = 154) | 15.2 ± 1.3 | 15.1 ± 1.3 | **-0.6** | **0.016^a^** |
| ALM, kg (n = 154) | 17.9 ± 2.3 | 17.6 ± 2.3 | **-1.5** | **<0.001^a^** |
| ALMI, kg/m^2^ (n = 154) | 6.6 ± 0.7 | 6.5 ± 0.6 | **-1.5** | **<0.001^a^** |
| Right leg lean mass, kg (n = 154) | 6.7 ± 0.9 | 6.6 ± 0.9 | **-1.8** | **<0.001^a^** |

Values are given as mean ± SD. ALM, appendicular lean mass; ALMI, appendicular lean mass index; BMI, body mass index; E_2_, estradiol; FSH, follicle stimulating hormone; LBM, lean body mass; LBMI, lean body mass index; MET, metabolic equivalent; MVPA, moderate-to-vigorous physical activity. ^a^ paired t-test, ^b^ Wilcoxon Signed rank test,

^X^ accelerometer-measured, ^XX^ self-reported. Significant results (*P* ≤ 0.050) are shown in bold.
