# Supplementary material for: Role of Menopausal Transition and Physical Activity in Loss of Lean and Muscle Mass: A Follow-Up Study in Middle-Aged Finnish Women
Source: J Clin Med. 2020 May 23;9(5):1588. doi: 10.3390/jcm9051588 (PMC7290663; doi:10.3390/jcm9051588)
Supplement: Supplementary file 1 [file jcm-09-01588-s001.zip › S3_JCM.docx]

**Table S3.** Characteristics of the participants who went through natural menopausal transition (non-HT users).

|  | Baseline  n = 197 | Final follow-up  n = 197 | Difference % | *P* |
| --- | --- | --- | --- | --- |
| Age, y | 51.8 ± 1.9 | 53.1 ± 1.9 | **+2.6** | **<0.001**^a^ |
| Body mass, kg | 69.8 ± 11.4 | 70.5 ± 11.7 | **+1.0** | **<0.001**^b^ |
| BMI, kg/m^2^ | 25.6 ± 4.1 | 25.9 ± 4.2 | **+1.0** | **<0.001**^b^ |
| E_2_, nmol/L | 0.34 ± 0.28 | 0.21 ± 0.15 | **-39** | **<0.001**^b^ |
| FSH, IU/L | 36.9 ± 20.8 | 71.3 ± 28.3 | **+93** | **<0.001**^b^ |
| Physical activity |  |  |  |  |
| MVPA, min/day^X^ (n = 153) | 51.0 ± 29.3 | 49.3 ± 23.7 |  | 0.693^b^ |
| MET-hours/day^XX^ (n = 195) | 4.3 ± 4.0 | 4.6 ± 3.7 | **+5.5** | **0.043**^b^ |
| DXA-measurements |  |  |  |  |
| LBM, kg (n = 188) | 41.6 ± 4.3 | 41.4 ± 4.4 | **-0.5** | **0.027**^a^ |
| LBMI, kg/m^2^ (n = 188) | 15.3 ± 1.3 | 15.2 ± 1.3 | **-0.5** | **0.026**^a^ |
| ALM, kg (n = 188) | 17.9 ± 2.2 | 17.7 ± 2.2 | **-1.3** | **<0.001**^a^ |
| ALMI, kg/m^2^ (n = 188) | 6.6 ± 0.6 | 6.5 ± 0.6 | **-1.3** | **<0.001**^a^ |
| Right leg lean mass, kg (n = 188) | 6.8 ± 0.8 | 6.7 ± 0.8 | **-1.2** | **0.002**^a^ |
| Computed tomography |  |  |  |  |
| Absolute muscle area, cm^2^  (n = 68) | 166.4 ± 9.8 | 164.9 ± 10.4 | **-0.9** | **0.003^a^** |
| Relative muscle area, % (n = 67)* | 69.9 ± 5.5 | 69.2 ± 6.0 | **-0.9** | **0.001**^a^ |

Values are given as mean ± SD. ALM, appendicular lean mass; ALMI, appendicular lean mass index; BMI, body mass index; E_2,_ estradiol; FSH, follicle stimulating hormone; LBM, lean body mass; LBMI, lean body mass index; MET, metabolic equivalent; MVPA, moderate-to-vigorous physical activity. ^a^ paired t-test, ^b^ Wilcoxon Signed rank test,  ^X^ accelerometer-measured, ^XX^ self-reported, * n = 67: because of a technical failure in one CT scan, relative muscle area could not be calculated. Significant results (*P* ≤ 0.050) are shown in bold.
