# Supplementary material for: Role of Menopausal Transition and Physical Activity in Loss of Lean and Muscle Mass: A Follow-Up Study in Middle-Aged Finnish Women
Source: J Clin Med. 2020 May 23;9(5):1588. doi: 10.3390/jcm9051588 (PMC7290663; doi:10.3390/jcm9051588)
Supplement: Supplementary file 1 [file jcm-09-01588-s001.zip › S4_JCM.docx]

**Table S4.** Characteristics of the participants who started using HT during follow-up.

|  | Baseline  n = 37 | Final follow-up  n = 37 | Difference  % | *P* |
| --- | --- | --- | --- | --- |
| Age, y | 50.6 ± 1.8 | 52.1 ± 2.0 | **+3.0** | **<0.001**^b^ |
| Body mass, kg | 70.1 ± 10.6 | 70.3 ± 11.2 |  | 0.210^b^ |
| BMI, kg/m^2^ | 25.4 ± 3.5 | 25.5 ± 3.6 |  | 0.230^b^ |
| E_2_, nmol/L | 0.30 ± 0.21 | 0.41 ± 0.27 |  | 0.054^b^ |
| FSH, IU/L | 31.5 ± 24.9 | 44.2 ± 31.9 | **+40** | **0.047**^b^ |
| Physical activity |  |  |  |  |
| MVPA, min/day^X^ (n = 20) | 58.1 ± 28.7 | 52.6 ± 23.2 |  | 0.526^b^ |
| MET-hours/day^XX^ (n = 36) | 5.1 ± 3.6 | 5.3 ± 3.6 |  | 0.879^b^ |
| DXA-measurements |  |  |  |  |
| LBM, kg (n = 35) | 42.1 ± 4.6 | 41.9 ± 4.5 |  | 0.405^a^ |
| LBMI, kg/m^2^ (n = 35) | 15.3 ± 1.3 | 15.2 ± 1.2 |  | 0.387^a^ |
| ALM, kg (n = 35) | 18.3 ± 2.5 | 18.2 ± 2.3 |  | 0.589^b^ |
| ALMI, kg/m^2^ (n = 35) | 6.6 ± 0.7 | 6.6 ± 0.6 |  | 0.594^a^ |
| Right leg lean mass, kg (n = 35) | 6.9 ± 1.0 | 6.8 ± 0.8 |  | 0.503^a^ |
| Computed tomography |  |  |  |  |
| Absolute muscle area, cm^2^ (n = 9) | 170.8 ± 8.0 | 168.3 ± 8.0 | **-1.5** | **0.021**^b^ |
| Relative muscle area, % (n = 9) | 67.8 ± 5.9 | 66.8 ± 5.8 |  | 0.052^a^ |

Values are given as mean ± SD. ALM, appendicular lean mass; ALMI, appendicular lean mass index; BMI, body mass index; E_2,_ estradiol; FSH, follicle stimulating hormone; LBM, lean body mass; LBMI, lean body mass index; MET, metabolic equivalent; MVPA, moderate-to-vigorous physical activity. ^a^ paired t-test, ^b^ Wilcoxon Signed rank test, ^X^ accelerometer-measured, ^XX^ self-reported. Significant results (*P* ≤ 0.050) are shown in bold.
