# Supplementary material for: Role of Menopausal Transition and Physical Activity in Loss of Lean and Muscle Mass: A Follow-Up Study in Middle-Aged Finnish Women
Source: J Clin Med. 2020 May 23;9(5):1588. doi: 10.3390/jcm9051588 (PMC7290663; doi:10.3390/jcm9051588)
Supplement: Supplementary file 1 [file jcm-09-01588-s001.zip › S5_JCM.docx]

**Table S5.** GEE-model with accelerometer-measured moderate-to-vigorous physical activity as a measure of physical activity (MVPA, min/day).

|  | **Model 1** | | **Adjusted model** | |
| --- | --- | --- | --- | --- |
|  | ***B*** | ***P - value*** | ***B*** | ***P - value*** |
| LBM  Menopausal status  Use of HT  Use of progestogen  Follow-up time  MVPA  Age | -0.242  3.6*10^-5^  1.324  0.000  -  - | **0.013**  0.980  0.036  0.705  -  - | -0.231  0.000  1.371  0.000  0.008  0.073 | **0.016**  0.941  **0.034**  0.676  **0.028**  0.662 |
| LBMI  Menopausal status  Use of HT  Use of progestogen  Follow-up time  MVPA  Age | -0.089  0.000  0.278  0.000  -  - | **0.012**  0.730  0.125  0.583  -  - | -0.086  0.000  0.365  0.000  0.002  0.114 | **0.015**  0.832  **0.047**  0.313  0.079  **0.016** |
| ALM  Menopausal status  Use of HT  Use of progestogen  Follow-up time  MVPA  Age | -0.304  0.001  0.696  0.000  -  - | **< 0.001**  0.196  **0.022**  0.471  -  - | -0.292  0.001  0.690  0.000  0.009  0.009 | **< 0.001**  0.160  **0.026**  0.540  **< 0.001**  0.916 |
| ALMI  Menopausal status  Use of HT  Use of progestogen  Follow-up time  MVPA  Age | -0.113  0.000  0.169  0.000  -  - | **< 0.001**  0.231  **0.049**  0.329  -  - | -0.108  0.000  0.197  0.000  0.003  0.042 | **< 0.001**  0.165  **0.024**  0.212  **< 0.001**  0.085 |
| Right leg lean mass  Menopausal status  Use of HT  Use of progestogen  Follow-up time  MVPA  Age | -0.119  0.001  0.265  0.000  -  - | **< 0.001**  0.124  **0.021**  0.399  -  - | -0.114  0.001  0.261  0.000  0.004  0.002 | **< 0.001**  0.097  **0.025**  0.482  **< 0.001**  0.946 |
| Absolute muscle area*  Menopausal status  Use of HT  Follow-up time  MVPA  Age | -1.803  0.001  -0.001  -  - | **0.001**  0.826  0.871  -  - | -1.751  0.001  -0.001  0.018  0.071 | **0.001**  0.786  0.868  0.228  0.922 |
| Relative muscle area*  Menopausal status  Use of HT  Follow-up time  MVPA  Age | -0.007  -3.1*10^-5^  -3.5*10^-6^  -  - | **0.001**  0.184  0.864  -  - | -0.007  -2.8*10^-5^  -6.6*10^-6^  8.1*10^-6^  -0.002 | **0.001**  0.216  0.755  0.879  0.519 |

Model 1: adjusted for menopausal status, HT use in days, baseline use of progestogen and follow-up time in days. Adjusted model: adjusted for menopausal status, HT use in days, baseline use of progestogen follow-up time in days, MVPA-minutes/day and age at baseline. *Absolute and relative muscle cross-sectional areas were not adjusted for baseline progestogen use, as all the participants were non-users at baseline. ALM, appendicular lean mass; ALMI, appendicular lean mass index; HT, hormonal replacement therapy; LBM, lean body mass; LBMI, lean body mass index; MVPA, moderate-to-vigorous physical activity. Significant results (P ≤ 0.050) are shown in bold.
