# Supplementary material for: Role of Menopausal Transition and Physical Activity in Loss of Lean and Muscle Mass: A Follow-Up Study in Middle-Aged Finnish Women
Source: J Clin Med. 2020 May 23;9(5):1588. doi: 10.3390/jcm9051588 (PMC7290663; doi:10.3390/jcm9051588)
Supplement: Supplementary file 1 [file jcm-09-01588-s001.zip › S6_JCM.docx]

**Table S6.** Characteristics of biopsied participants.

|  | Baseline  n = 25 | Final follow-up  n = 25 | Difference  % | *P* |
| --- | --- | --- | --- | --- |
| Age, y | 52.2 ± 2.1 | 53.4 ± 1.9 | **+2.3** | **< 0.001**^a^ |
| BMI, kg/m^2^ | 25.9 ± 4.4 | 26.0 ± 4.6 |  | 0.313^b^ |
| E_2_, nmol/L | 0.32 ± 0.24 | 0.21 ± 0.14 |  | 0.067^b^ |
| FSH, IU/L | 42.6 ± 24.0 | 73.2 ± 21.7 | **+72** | **< 0.001**^b^ |
| Physical activity |  |  |  |  |
| MVPA, min/day^X^ | 50.2 ± 27.4 | 48.3 ± 24.1 |  | 0.493^b^ |
| MET-hours/day^XX^ | 4.8 ± 3.3 | 4.5 ± 2.9 |  | 0.989^b^ |

Values are given as mean ± SD. BMI, body mass index; E_2,_ estradiol; FSH, follicle stimulating hormone; MET, metabolic equivalent; MVPA, moderate-to-vigorous physical activity. ^a^ paired t-test, ^b^ Wilcoxon signed ranks, ^X^ accelerometer-measured, ^XX^ self-reported. Significant results (*P* ≤ 0.050) are shown in bold.
